# Clinical and Immunological Changes of Immunotherapy in Patients with Atopic Dermatitis: Randomized Controlled Trial

**DOI:** 10.5402/2012/183983

**Published:** 2012-03-07

**Authors:** Jorge Mario Sánchez Caraballo, Ricardo Cardona Villa

**Affiliations:** ^1^Group of Clinical and Experimental Allergy, University of Antioquia, Medellin, Colombia; ^2^Foundation for the Development of Medical and Biological Sciences (FUNDEMEB), Cartagena, Colombia; ^3^Institute for Immunological Research, University of Cartagena, Cartagena, Colombia; ^4^IPS Universitaria Sede Ambulatoria, Universidad de Antioquia Carrera 51A No. 62-42, Medellin, Colombia

## Abstract

*Background.* Immunotherapy has proven to be an useful tool in the management of allergic respiratory diseases; however, little has been studied in atopic dermatitis. *Objective.* To evaluate the clinical and immunological impact of immunotherapy with mites allergen extracts in atopic dermatitis. *Methods.* Patients with atopic dermatitis were assigned with computer-generated randomization to either of the following groups: (a) controls received only topical treatment with steroids and/or tacrolimus and (b) actively treated patients received topical treatment plus immunotherapy. Levels of serum total IgE, mites-specific IgE and IgG4 were assessed at study start and after one year of immunotherapy. *Results.* 31 patients in the active group and 29 in the control group completed the study. Symptoms and medication scores were significantly reduced in the active group after six months. Three patients in the control group showed new sensitizations to mites, while 3 patients in the active group showed neosensitization to shrimp with negative oral food challenge. We observed significant increase of mites-specific IgG4 levels in active group. *Conclusion.* Specific allergen immunotherapy induced a tolerogenic IgG4 response to mite allergens associated with favorable clinical effects in atopic dermatitis patients.

## 1. Introduction

Atopic dermatitis (AD) is an inflammatory skin disease characterized by pruritus, lichenified plaques, and family history of atopy. Good skin hydration and topical steroids are the keystone for symptomatic control. However, chronic steroid use is frequently associated with adverse effects. It is clear that Th1 immune response plays an important role in the development of AD, but allergens and Th2 immune response are the main cause of both initiation and exacerbation of AD [1].

At present, immunotherapy represents the only therapy for allergic diseases by targeting sensitization itself. Its beneficial clinical effects have been widely demonstrated in asthma, rinitis, and hymenoptera allergy in terms of both symptoms and medication scores [2, 3]. Some studies have shown that immunotherapy could be an option treatment in patients with AD; however there are few studies evaluating the immunological changes and the reduction of topical steroids in patients with AD [4, 5].

The aim of this study was to evaluate the immunomodulator and the clinical effect of the immunotherapy in the reduction of symptoms and topical drugs, in a group of patients with atopic dermatitis from a tropical city (Medellin Colombia).

## 2. Methods

### 2.1. Study Design (Figure 1)

This is an open label, controlled, randomized study. Patients were enrolled during September 2009 to January 2010. After a baseline, patients were stratified according to sensitization pattern in mono- and poly-sensitized and randomized to receive immunotherapy and pharmacotherapy (active group) or only pharmacotherapy (control group). The use of concomitant medications as emollients and topical and systemic drugs was permitted in both groups according to clinical evolution of each patient and was regularly registered. All patients were informed about general measures to reduce indoor allergens concentrations and to avoid possible aggravating factors (cigarette smoke, irritant volatile particles). 

### 2.2. Patients

Patients over 3 years of age with clinical history of AD for more than 2 years, IgE sensitization to *Dermatophagoides farinae (Der f)* and *Dermatophagoides pteronyssinus (Der p), *and *Scoring of Atopic Dermatitis* (SCORAD) [6] over 15 at the beginning of the study were selected. Exclusion criteria were administration of immune suppressors or biological agents in the last three months; significant improvement of symptoms in the last 6 months before enrollment; systemic diseases that contraindicated the use of immunotherapy [3].

Each patient was diagnosed according to Hanifin and Rejka criteria [7] and severity was assessed with SCORAD scale at the baseline and during the followup. Atopic dermatitis was ranked as severe, moderate, or mild if SCORAD scored over 40, 15, and 39 or below 15, respectively. Additionally, each patient or their parents answered a subjective evaluation consisting of 3 questions assessing general perception (Subjective Score SS) [4], SS ranging from 0 (no symptoms at all) to 10 (very severe symptoms); the average score of the three questions was expressed as a percentage. SCORAD and SS were repeated every three months during the followup.

### 2.3. Sensitization to Inhalant and Food Allergens

Sensitization was evaluated with prick test before and after one year with immunotherapy [8] using a panel of allergenic extracts: mites (*Der f, Der p *and *Blomia tropicalis (Blo t)*), pet's dander (cat and dog), textile fibers (cotton and wool), fungi (*Asperllilus fumigatus*,* Cladosporium*), excrement and pens from canary, dove and parakeet, insects (cockroach, mosquito, and ant), and pollen (group herbs, cereals, flowers, grasses, and trees). All this biological extracts were provided by Laboratories Leti Madrid, España.

Prick test with shrimp, egg (whole egg, egg white, egg yolk, ovomucoid (Gal d 1) and ovalbumin (Gal d 2)) and milk (Casein and total milk) using standardized extracts (Laboratories Leti Madrid, España) and fresh products was been performed in all patients. When a patient had suspected of allergy to other food, this was included in the prick test.

### 2.4. Total IgE and Specific IgE and IgG4 for *Der p* and *Der f*

We take a serum sample from patients who consented. Serum samples from 10 patients in the active group and 10 from control group were taken; 5 patients in each group were monosensitized and 5 polysensitized. Serum samples were collected at baseline and after one year of treatment. Serum levels of *Der f* and *Der p* specific IgE were measured using a flouroenzyme immunoassay (Phadia ImmunoCap System, Uppsala, Sweden). Sera yielding specific IgE levels above 100 IU/mL were preliminarily diluted (1 : 5) to maintain the test within the dynamic range. IgG4 was measured using ELISA technique. Briefly microtiter plates coated with *Der f* and *Der p* were blocked with bovine serum albumin and then incubated with serum samples from atopic dermatitis patients. Monoclonal antibodies against IgG4 were added followed by biotinylated rabbit anti-mouse antibody and horse radish peroxidase-conjugated streptavidine. Tetramethyl benzidine (TMB) was used as substrate.

### 2.5. Single-Blind Placebo-Controlled Food Challenge (SBPCFC)

SBPCFC were due in patients with a strong clinical suspected of food allergy and with positive prick test for milk, shrimp, or egg according to international recommendations [9].

### 2.6. Immunotherapy

Subcutaneous immunotherapy with depigmented polymerized mites extract (0,5 mL *Der f*/*Der p*, 50DPP, laboratories Leti Madrid España) was administrated monthly. Mite allergen extracts were administered in two refracted doses of 0,2 and 0,3 mL at first injection, and in single 0,5 mL doses in subsequent monthly injections. A 30 min observation time was required after each injection, for observing and counteracting possible side effects. Patients or patients' parents were instructed to identify and report any local or systemic reaction.

### 2.7. Pharmacologic Treatment

Oral antihistamines, emollients, topical steroids, and tacrolimus were administrated in staggered steps according the severity of symptoms [6]. In case of mild exacerbation topical steroids could be prescribed for short periods (less than 30 days) followed by topical tacrolimus. In case of severe exacerbation oral steroids were permitted for 7 days. Doses, frequency of use, and relative potency of drugs were registered and a point scale was constructed according to these variables taking in consideration the recommendations of ETFAD/EADV eczema task force 2009 [6].

AD severity was evaluated after one year since study start. The following clinical levels of control were defined. “Good controls” applied to patients with no skin exacerbations for at least 6 months, topical steroid use, and SCORAD reduction >40% versus baseline. “Regular Control” applied to patients with <2 skin exacerbations in the last 6 months and reduction >20% in topical steroids and >40% in SCORAD, respectively. “Poor Control” applied when neither were met.

### 2.8. Ethics Considerations

The study was approved by the ethics committee of University of Antioquia and Clínica Leon XIII (Medellin Colombia). All patients or their parents gave written informed consent.

### 2.9. Statistical Analysis

Statistical Analysis was performed with IBM SPSS statistics 19.0 for Windows. Efficacy of treatment, reduction of drugs, wheal sizes, total IgE, and specific IgE and IgG4 were compared between both groups using the Mann-Whitney test. Because the effect of immunotherapy is detectable after several months of treatment, evaluation of effectiveness was performed based on protocol analysis; calculated values were expressed as means and standard deviations of the mean. Proportions were analyzed using contingency tables, and the Fisher exact test was conducted. *P* < 0.05 was considered statistically significant. For the primary outcome (clinical response), a sample size of 58 subjects would provide a statistical power of 80% to detect a mean difference of 30%.

## 3. Results

### 3.1. Baseline Description of Patients

**Figure 1:**
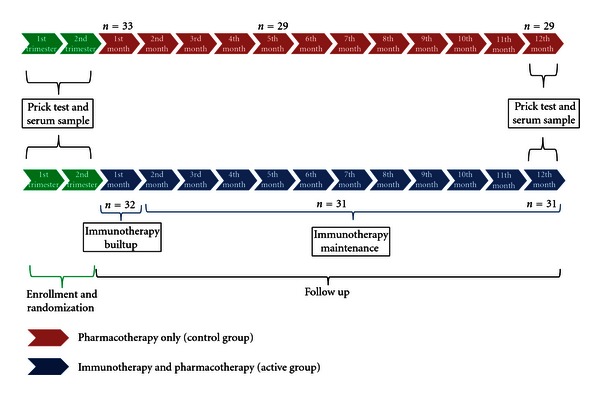
Study design: in both groups, patients were called monthly to evaluated use of medications and clinical control of symptoms. Each 3 months a complete clinical evaluation was due. Immunotherapy injection was due each month.

Sixty-five patients were included; 4 patients in the control group and 1 in the active group were excluded because they moved to other cities (Figure 1). Sixty patients between 3 and 25 years finalized the study. All patients had sensitization to *Der f* and *Der p*; 14 to dog dander; 3 to pollen grains; 5 to *Solenopsis invicta* (fire ant). We found 22 sensitizations to food, 9 to milk, 7 to egg and 6 to shrimp. In total, 29 patients were polysensitized (Table 1).

### 3.2. Significant Reduction in Objective (SCORAD) and Subjective (SS) Symptoms Scale

**Figure 2:**
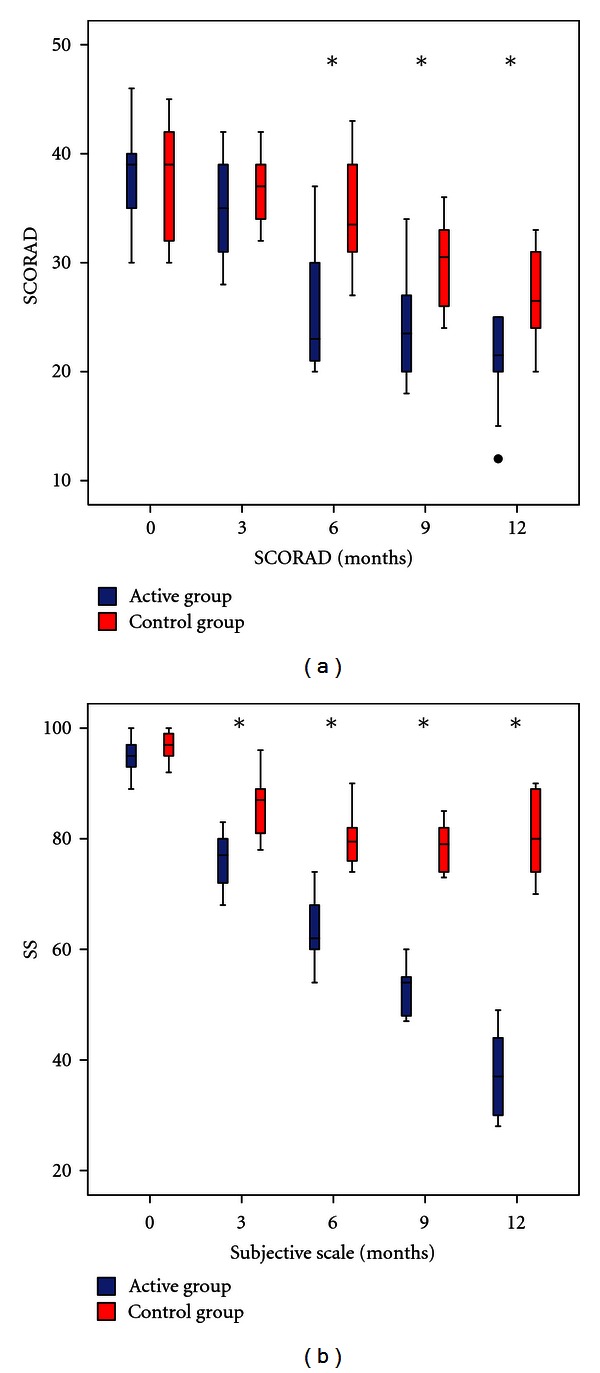
Change in symptoms evaluated with objective and subjective scales at different time points. Blue: Active group. Red: Control Group. SCORAD: Scoring Atopic Dermatitis, VAS: Analog Visual Scale, SS: Subjective Scale. *P* ≤ 0.05; *n* = 60; Mann-Whitney *U* test, Wilcoxon test.

Most patients showed an important reduction in symptoms. After 6 months, active group had a significant improvement over control group in SCORAD (*P* = 0,03) and SS (*P = *0,01). We did not observed significant differences according to age or gender. Nine patients in the active group and 5 in the control group had a SCORAD >40; after one year 6 of these patients in the active group had a significant improvement compared to only 2 in the control group (Figure 2).

### 3.3. Significant Reduction in Steroids and Topical Tacrolimus

**Figure 3:**
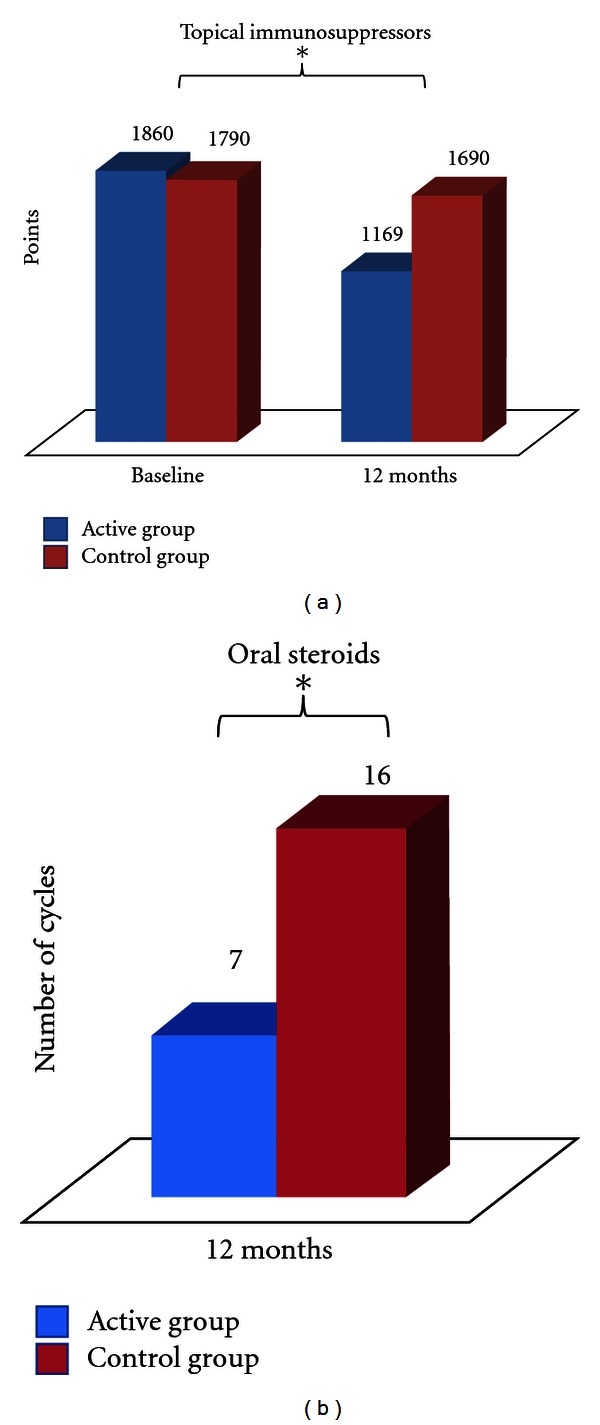
Use of topical immunosuppressors in baseline and after 12 months of treatment. Points were assigned according to dose, frequency of use, and relative potency of the steroid used. Blue: Active Group. Red: Control Group. *P ≤ 0.05;  *n* = 60; Mann-Whitney *U* test, Wilcoxon test.

**Figure 4:**
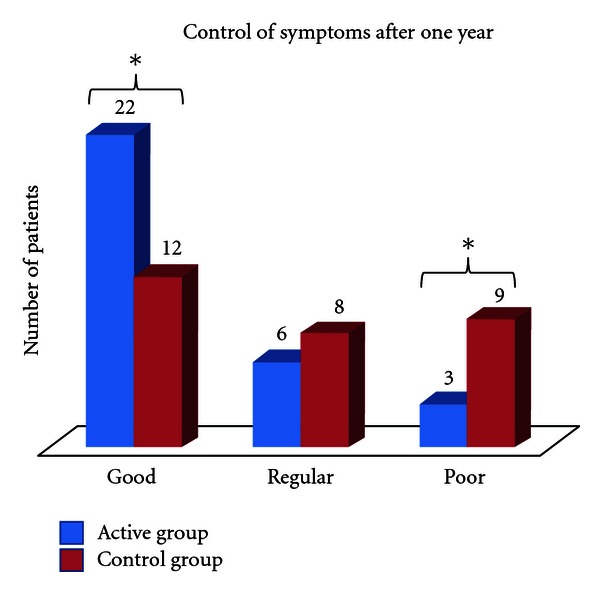
Control of atopic dermatitis according to improvement in SCORAD and reduction in pharmacology treatment in the last 6 months of the study. Blue: Active Group. Red: Control Group. *P ≤ 0.05;  *n* = 60; Mann-Whitney *U* test, Wilcoxon test.

After 1 year of followup a reduction in use of topical steroids and tacrolimus was presented in the active group compared with control group (*P* = 0,02). Active group also required less oral steroid cycles (*P* = 0,02) (4 patients in immunotherapy and 12 in control group) (Figure 3). The frequency of antihistamines and emollients had no significant change between groups. twenty-two patients with immunotherapy and only 11 in control group had good control after one year (*P* < 0.01) (Figure 4). 

### 3.4. Change in Sensitization Pattern in Both Groups after One Year

After one year, change in pattern sensitization in both groups were observed (Table 2). In the prick test, the active group had a reduction in the diameter of the wheal for *Der f* and *Der p* and the control group had a small increase but without significant difference between groups. Three patients in the active group turned prick test with allergen mite extracts to negative and three displayed new sensitizations to shrimp and one cockroach. One of the patients with new sensitization to shrimp ate shrimp for the first time during immunotherapy and continued to do so without symptoms or association with exacerbations. The others two patients were children that never ate shrimp, lobster, or clams. In the controls the new sensitizations to different allergens appeared after 1 year, namely, three patients scored positive to *Blomia tropicalis*, one patient to grass, and two to dog dander. 

### 3.5. Total IgE and Specific IgE and IgG4 Levels

**Figure 5:**
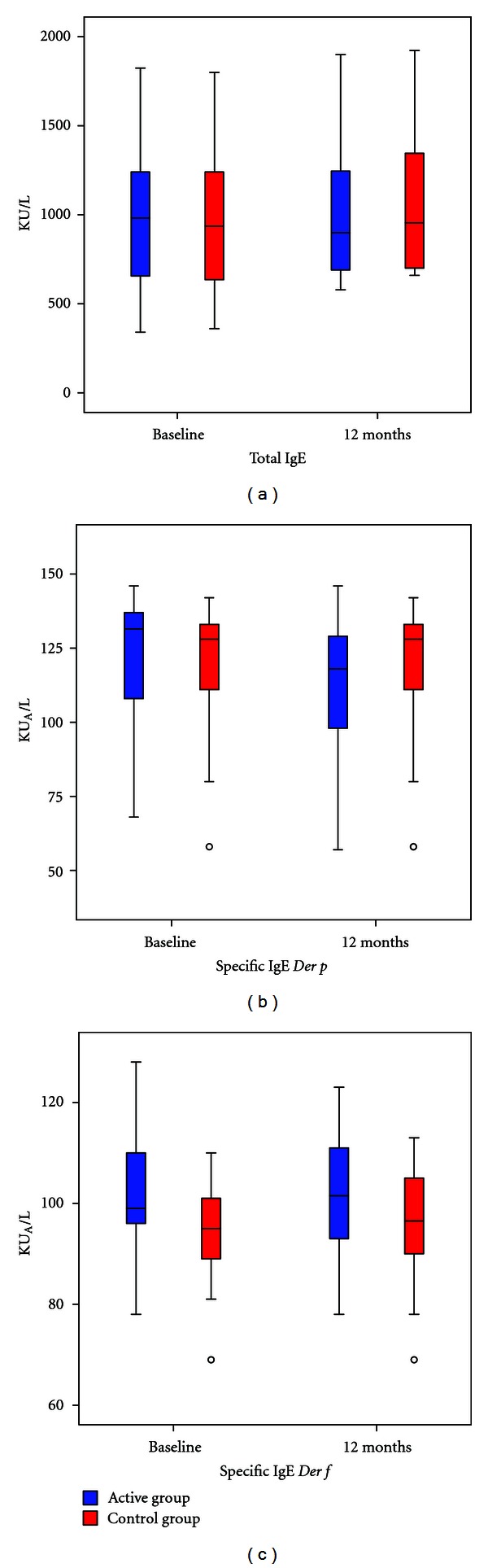
Change in IgE total (a) and specific IgE for *Der p* (b) and *Der f* (c) between active and control group before and after one year. Blue: Active group. Red: Control Group. *P ≤ 0.05.

**Figure 6:**
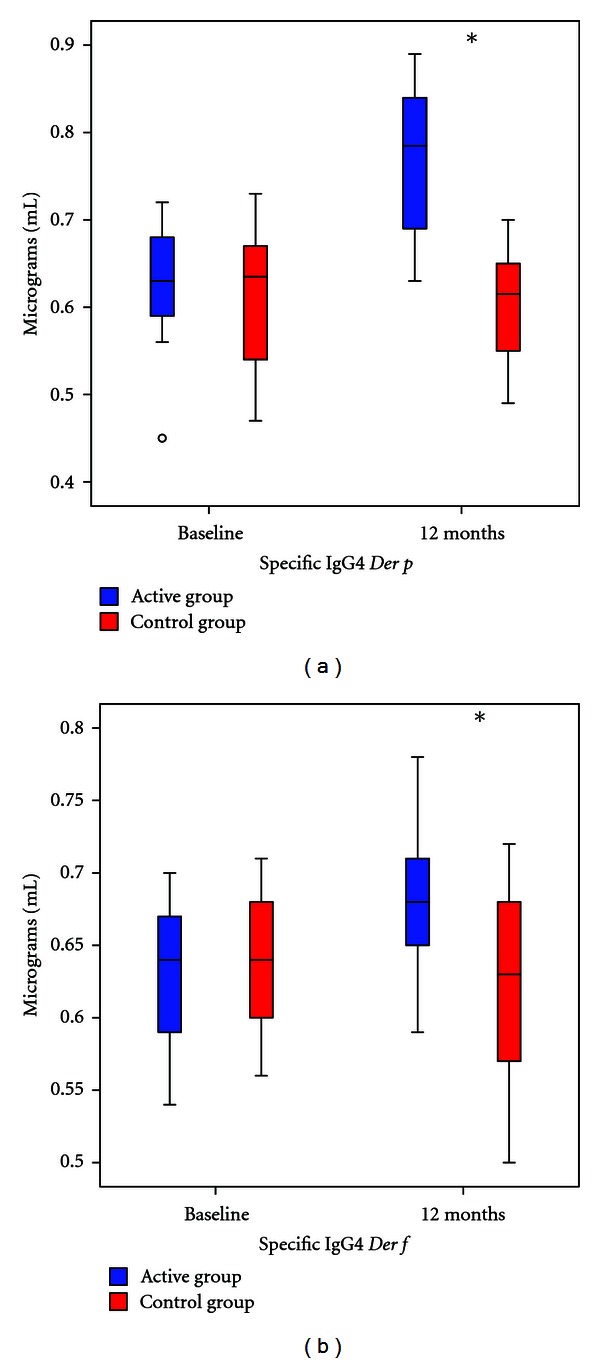
Change in specific IgG4 for *Der p* (b), and *Der f* (c) between active and control group before and after one year. Blue: Active Group. Red: Control Group. *P ≤ 0.05.

There were no significant differences in total IgE and specific IgE for *Der p* and *Der f* between groups (Figure 5). *Der p* and *Der f* specific IgG4 levels were significantly increased in the group with immunotherapy (*P* < 0,05). The increase of IgG4 was more significant in the 5 monosensitized patients from the active group (*P* = 0,01) (Figure 6).

### 3.6. Single-Blinded Placebo-Controlled Food Challenge (SBPCFC)

At the beginning of the study 26 patients had a self-reported adverse reaction after eating some food but in 24 of these patients the clinical history was not clear and had a negative prick test. Only 2 patients had a clinical history suggestive of food allergy and a positive prick test with the suspected food (egg and shrimp) but both patients had a negative oral food challenge.

The three patients in the active group with neosensitization to shrimp showed no symptoms after oral challenge.

### 3.7. Comorbidities

Forty-seven patients had asthma or rhinitis and were treated according to GINA and ARIA guidelines. All patients presented a significant reduction of respiratory symptoms after 3 months; active group had an important reduction in inhaled steroids over control group (data not shown).

### 3.8. Immunotherapy Adverse Effects

In the first three months of treatment, sixteen local immediate reactions were observed in eleven patients, whereas no systemic reactions were recorded.

## 4. Discussion

Atopic dermatitis typically begins in early infancy and by age fifteen virtually disappears. However, in a group of patients AD persists with a severe impact of the quality of life, due not only to the disease itself but also to the side effects of required pharmacological therapies, which are based on oral steroids, cyclosporine, azathioprine, methotrexate, and other immunosuppressors [10].

Some studies have reported that allergen immunotherapy is well tolerated and produces significant clinical response in most of patients with atopic dermatitis after 2 to 12 months, but in most of these studies there is no control group and the requirement of controller medications is not evaluated [11–14]. We observed that patients with immunotherapy presented an important reduction of symptoms and affected surface body area and used of topical immunosuppressors and oral steroids after 6 months of treatment. This clinical response in the active group was observed independently of patient sensitization status (mono- or polysensitized). These results indicate that immunotherapy is an effective treatment with important clinical repercussion in patients with atopic dermatitis and may reduce the risk of adverse effects from prolonged use of oral and topical immunosuppressors. Because this was an open study, in order to avoid placebo effect, we considered significant clinical response only in those patients that presented changes in subjective and objective scales greater than 30%, and reduction no less than 20% in topical steroids and tacrolimus. Even with these high cutoffs, we found that most of patients with immunotherapy had a very good symptom control and most of them did not require oral steroids in the last six months of followup.

Bussmann et al. found a significant reduction in mites-specific IgE from patients with atopic dermatitis receiving immunotherapy and this was in turn correlated with clinical improvement especially in patients with severe presentations [11]. In a multicentre study the high impact of immunotherapy was found in patients with SCORAD > 40 [13], in agreement with our results. Immunological changes such as the reduction of chemotactic mediators and changes in specific IgE and IgG4 had been studied in atopic dermatitis patients receiving immunotherapy with controversial results [11, 15]. We observed a significant increase in mites-specific IgG4 in patients with immunotherapy after 1 year of treatment and a small but not significant reduction in hives diameters from skin prick test and specific IgE levels for *Der p*. The monosensitized group receiving immunotherapy had the highest increase in mite-specific IgG4; however this was not correlated with a greater clinical response when compared to the polysensitized group with immunotherapy.

Another interesting finding in our study was the change in sensitization pattern between control and active group; in the control group we observed neosensitization to allergen sources with little evidence of cross-reactivity like dog dander, pollen grains, and *Blomia tropicalis*. In the active group we observed neosensitization in 2 patients to shrimp and 1 patient to shrimp and cockroach, both invertebrates with known cross-reactivity between them and with mites [16–18]. These food neosensitizations in the active group was not associated with clinical symptoms and oral challenge was negative in these patients. In our opinion, all these changes support a positive immunomodulator effect of immunotherapy in atopic dermatitis analogous to those observed in other allergic diseases. Immunotherapy in asthma and rhinitis has proven to be useful to increase an immunoregulatory response and avoiding new sensitizations [19–23] but some articles report new sensitizations with shrimp [24]. To our knowledge this effect has not been studied in patients with atopic dermatitis. One explanation for the neosensitization with shrimp in 3 patients in the active group could be a direct or indirect contact to shrimp, cockroach, or another cross-reactive allergic source during followup. The presence of a highly cross-reactive, homologous allergen component, such as tropomyosin, in mites and shrimps allergen extracts, might account for the spreading of sensitization to the latter (though in the absence of clinical signs). Notably, SBPCFC testing with shrimp extracts, which yielded negative results, excluded relevant clinical outcomes associated with the acquisition of these new sensitizations. Since sensitization with invertebrates only occurred in active group, this raises the possibility that sensitization occurred by cross-reactivity between shrimp proteins and mite's proteins present in the extract for immunotherapy [24, 25]. Patients with allergic diseases have reactions to specific mite proteins; when they receive immunotherapy with mite, extracts have a high exposition with a high number of proteins with potential allergenicity. The risk of new sensitization with these proteins could be high in patients with atopic dermatitis due to their high polyclonal IgE response and severe skin inflammation. However, we used depigmented and glutaraldehyde-modified allergen extracts. This modifications produces allergens with decreased IgE-binding capacity [26] which have also been proven to be safe and with good clinical efficacy [27–29]. In patients with shrimp neosensitization, we did an oral challenge with shrimp and we did prick tests with the mites extract used for immunotherapy; both tests were negative in the three patients, This suggests that this sensitization is clinically irrelevant and a relation between immunotherapy and neosensitization to shrimp is unlikely. All three patients presented significant improvement in atopic dermatitis and in their allergic comorbidities.

One patient with milk sensitization did not display clinical signs of milk allergy and became skin prick test negative after one year. This behavior is typical of the natural history of milk and egg allergy in children [30, 31]. We observed early local reactions after injection of immunotherapy in some patients, but they occurred only during the first applications and none of our patients withdrew from the study or required hospitalization because severe adverse reactions with immunotherapy.

In conclusion, our results suggest that allergen-specific immunotherapy can induce a tolerogenic IgG4 response to mite allergens, is clinically effective and safe, and can be proposed as an important tool in the management of atopic dermatitis. Further studies are needed to assess the impact of immunotherapy in the prevention of new sensitizations in atopic dermatitis patients.

## Figures and Tables

**Table 1:** Characteristics of patients. SCORAD: Scoring Atopic Dermatitis, SS: Subjective Score.

Baseline characteristics	Groups	
	Active (%)	Control (%)
Patients number	31 (100)	29 (100)
Age	8 years (3–24)	8 years (3–25)
Gender (female)	16 (52)	14 (48)
Atopic Dermatitis	31 (100)	29 (100)
Asthma/Rhinitis	25 (81)	22 (76)
Monosensitization	16 (52)	15 (52)
Polysensitization	15 (48)	14 (48)
SCORAD (points)	38 (30–46)	38 (30–40)
SS%	95 (89–100)	98 (97–100)

**Table 2:** Prick test.

Extract	Active group		Control group	
	Baseline	12 months	Baseline	12 months
*Der f*	31	28	29	29
*Der p*	31	28	29	29
*Blo t*	12	12	9	12
Dog dander	7	7	7	9
Pollen grains	2	2	1	2
Cockroach	0	1	0	0
*Solenopsis Invicta*	3	3	2	2
Milk	5	4	4	4
Egg	4	4	3	3
Shrimp	3	6	3	3
